# Microbiome characterization of patients with Crohn disease and the use of fecal microbiota transplantation: A review

**DOI:** 10.1097/MD.0000000000041262

**Published:** 2025-01-24

**Authors:** Shiju Chen, Daya Zhang, Da Li, Fan Zeng, Chen Chen, Feihu Bai

**Affiliations:** a Graduate School, Hainan Medical University, Haikou, China; b Department of Gastroenterology, The Second Affiliated Hospital of Hainan Medical University, Haikou, China; c The Gastroenterology Clinical Medical Center of Hainan Province, Haikou, China.

**Keywords:** Crohn disease, fecal microbiota transplantation, microbiome characterization

## Abstract

Inflammatory bowel disease is a chronic inflammatory condition predominantly affecting the intestines, encompassing both ulcerative colitis and Crohn disease (CD). As one of the most common gastrointestinal disorders, CD’s pathogenesis is closely linked with the intestinal microbiota. Recently, fecal microbiota transplantation (FMT) has gained attention as a potential treatment for CD, with the effective reestablishment of intestinal microecology considered a crucial mechanism of FMT therapy. This article synthesizes the findings of population-based cohort studies to enhance our understanding of gut microbial characteristics in patients with CD. It delves into the roles of “beneficial” and “pathogenic” bacteria in CD’s development. This article systematically reviews and compares data on clinical response rates, remission rates, adverse events, and shifts in bacterial microbiota. Among these studies, gut microbiome analysis was conducted in only 7, and a single study examined the metabolome. Overall, FMT has demonstrated a partial restoration of typical CD-associated microbiological alterations, leading to increased α-diversity in responders and a moderate shift in patient microbiota toward the donor profile. Several factors, including donor selection, delivery route, microbial state (fresh or frozen), and recipient condition, are identified as pivotal in influencing FMT’s effectiveness. Future prospective clinical studies with larger patient cohorts and improved methodologies are imperative. In addition, standardization of FMT procedures, coupled with advanced genomic techniques such as macroproteomics and culture genomics, is necessary. These advancements will further clarify the bacterial microbiota alterations that significantly contribute to FMT’s therapeutic effects in CD treatment, as well as elucidate the underlying mechanisms of action.

## 1. Introduction

Inflammatory bowel disease (IBD) is a chronic inflammatory disorder primarily affecting the intestinal tract, encompassing both ulcerative colitis (UC) and Crohn disease (CD). The incidence of CD has progressively increased over the years, with a prevalence rate rising from 79.5 to 84.3 per 100,000 people between 1990 and 2017.^[1,2]^ This escalating prevalence imposes significant economic and healthcare burdens worldwide. Despite being a global disease in the 21st century, the pathogenesis of CD remains incompletely understood, and a standardized diagnostic gold standard is lacking. Diagnosis is predominantly based on clinical presentation, endoscopic manifestations, and histopathologic features. Currently, the disease remains incurable, and treatment options mainly involve 5-aminosalicylic acid, hormones, immunosuppressants, and biologics. However, many patients experience poor response to medication, along with potential side effects and drug dependency (e.g., hormones), posing significant therapeutic challenges. Recurrent, unhealing episodes are common, often leading to surgical interventions, albeit with a high rate of postoperative recurrence. Consequently, there is an urgent need for safer and more effective medications or novel treatment modalities for this disease.

In the past decade, researchers have increasingly focused on gut microbes. Dysbiosis of the intestinal microbiota is recognized as a contributing factor in the pathogenesis of CD. Studies have revealed significantly lower intestinal diversity in patients with CD compared with healthy controls, characterized by reduced levels of beneficial bacteria (which inhibit inflammation) and increased levels of harmful bacteria (which promote inflammation), accompanied by alterations in metabolic substances. To address this, various interventions have been explored, such as probiotics, prebiotics, synthetic metabolites, and fecal microbiota transplantation (FMT), aiming to improve the intestinal microecology of patients and modulate their immune systems. FMT has gained attention as a popular research focus, involving the transplantation of functional microbiota from a healthy donor into the patient’s gut through various routes within the gastrointestinal tract. The practice of fecal medicine dates back thousands of years.^[3]^ For instance, Ge Hong of the Eastern Jin Dynasty in ancient China described the use of dung juice in treating patients on the brink of death, including those with severe food poisoning, high fever, abdominal pain, and diarrhea.^[4]^

Nowadays, FMT has been utilized for >80 intestinal and extraintestinal diseases,^[5]^ including the following categories: infections, especially Clostridium difficile infections (CDIs)^[5]^; intestinal diseases, such as IBD^[6,7]^ and irritable bowel syndrome^[8]^; cirrhosis of the liver,^[9]^ hepatic encephalopathy,^[10]^ and other disorders related to disorders of the microbiota–gut–brain axis; autism,^[11]^ Parkinson disease,^[12]^ and other diseases related to disorders of the microbiota–gut–brain axis; and others include metabolic syndrome^[13]^ and tumors.^[14]^ The 3 most widely used diseases are CDI, IBD, and irritable bowel syndrome.^[5]^ Among them, FMT has demonstrated safety and efficacy in the treatment of recurrent CDI, with remission rates exceeding 90%, leading to its recommendation in multinational guidelines.^[15]^ FMT for the treatment of IBD and irritable bowel syndrome is also considered to be a promising therapeutic approach. In addition to this, FMT was found to improve Child-Turcotte-Pugh score, inhibit pro-inflammatory cytokine release, and reduce blood ammonia in cirrhotic patients in studies related to FMT and cirrhosis.^[5]^ Ding et al^[14]^ first reported that FMT offers the potential for the treatment of chronic refractory radiation enteritis. It was effective in improving the symptoms of diarrhea, rectal bleeding, abdominal/rectal pain, and fecal incontinence in patients with radiation enteritis.^[5]^ FMT has also achieved good efficacy in the treatment of refractory immune checkpoint inhibitor-associated colitis.^[16]^ This article provides a comprehensive summary of patients with CD-specific intestinal microbiota characteristics, as well as the effectiveness and safety of FMT in CD treatment and resultant microbiota changes. The aim is to advance our understanding of CD pathogenesis and the role of FMT as a therapeutic approach for CD.

## 2. Materials and methods

### 2.1. Studies related to differences in intestinal microbiota between CD and healthy people

The databases PubMed, Embase, and Cochrane were used to access studies from inception to January 2024, and search terms were devised ([Crohn Disease OR Crohn Disease] OR [Inflammatory Bowel Disease]) AND [Microbiota OR Microbiome OR Microflora]). Inclusion criteria for this review are given as follows: population-based observational prospective and retrospective studies, cohort studies, case-control studies, and randomized controlled trials; the articles included information on sample size and gut microbiota; the articles had to be written in English; detection of gut microbiota will be accomplished by stool samples and/or intestinal biopsies; and microbiota will be analyzed using metagenomic sequencing, 16S rRNA sequencing, polymerase chain reaction (PCR), real-time fluorescent quantitative PCR technology, or fluorescence in situ hybridization. Exclusion criteria are given as follows: studies in nonhuman subjects; reviews and case reports; studies were excluded if both CD and UC were reported, but they had not yet been separated; and this reported duplicate data. A total of 32 studies were included in this review for summary and discussion after combining the above nadir criteria (Table 1).

**Table 1:** Main gut bacteria changes in CD.

Phylum	Family	Genus	Alterations in genus/family	References	Species	Alterations in species	References
Bacteroidota	Bacteroidaceae	*Bacteroides*	↑	[17,18]	*B fragilis*	↑	[19]
			↓	[20,21,22]	*B uniformis*	↓	[20]
					*B thetaiotaomicron*	↓	[23]
	Rikenellaceae	*Alistipes*	↓	[24,25]	*A putredinis*	↓	[26]
	Prevotellaceae	*Prevotella*	↓	[21,22,24]			
Firmicutes	Lachnospiraceae	*Eubacterium*	↓	[20,24]	*E rectale*	↓	[27,28]
		*Anaerobutyricum*			*E/A hallii*	↓	[29]
		*Blautia*	↑	[30]	*B faecis*	↓	[20]
					*B obeum*	↓	[26,31]
		*Coprococcus*	↓	[21,24,32,33]			
		*Anaerostipes*	↓	[34,35]			
		*Roseburia*	↓	[21,24,36]	*R intestinalis*	↓	[19]
					*R inulinivoran*	↓	[20,30]
					*R hominis*	↓	[31]
	Clostridiaceae	*Clostridium*	↓	[37]	*C leptum*	↓	[17,18,38]
					*C lavalense*	↓	[20]
		*Faecalibacterium*	↓	[20,34,36]	*F prausnitzii*	↓	[17,19,20,27,29,39–41]
						↑	[42,43]
	Oscillospiraceae	*Flavonifractor*			*F plautii*	↑	[32]
		*Ruminococcus*	↓	[20,21,24,36]	*R gnavus*	↑	[21,26,29,31,35]
					*R albus*	↓	[27]
					*R callidus*	↓	[27]
					*R bromii*	↓	[27]
					*R torques*	↓	[20]
	Christensenellaceae		↓	[25,35,36,44]			
	Lactobacillaceae	*Lactobacillus*	↑	[17,45]			
			↓	[35,37]			
	Enterococcaceae	*Enterococcus*	↑	[24,45]			
Proteobacteria	Enterobacteriaceae	*Escherichia*	↑	[19,21,24,30,32,34]	*E coli*	↑	[17,19,21,41]
	Pasteurellaceae	*Actinobacillus*	↓	[21]			
	Desulfovibrionaceae	*Desulfovibrio*	↑	[46]			
			↓	[25]			
Actinobacteria	Bifidobacteriaceae	*Bifidobacterium*	↑	[17,20]	*B longum*	↓	[19]
			↓	[19,36]			
	Actinomycetaceae	*Actinomyces*	↑	[20]			
	Coriobacteriaceae		↑	[44]			
			↓	[28]			
Verrucomicrobia	Akkermansiaceae	*Akkermansia*	↓	[24]	*A muciniphila*	↓	[32,35,47]
Fusobacteria	Fusobacteriaceae	*Fusobacterium*	↑	[21,34,45]	*F nucleatum*	↑	[48]

↑ = higher, ↓ = lower.

### 2.2. Clinical studies of FMT in the treatment of CD

A comprehensive search was conducted using 3 databases (PubMed, Embase, and Cochrane) to identify relevant studies published between inception and January 2024, using specific search terms ([Crohn Disease] OR [Crohn disease] OR [Inflammatory Bowel Disease] AND [Fecal Microbiota Transplantation]). The inclusion criteria for studies are given as follows: conducted on patients with active CD, as indicated by a Harvey-Bradshaw index score ≥ 5, CD activity index score ≥ 150, pediatric CD activity index score ≥ 10, or IBD questionnaire score ≤ 170; adequately reported clinical efficacy and/or safety of FMT in patients with CD, including clinical remission, clinical response, minor adverse events (AEs), severe AEs, biochemical markers, and microbiological results; and cohort studies or randomized controlled trials. Exclusion criteria are given as follows: studies that reported both CD and UC without distinguishing between the 2; patients with CD with comorbidities such as CDI; and studies reporting duplicate data. We identified 13 clinical trials meeting the aforementioned criteria for inclusion in our review and subsequent discussion (Table 2).

**Table 2:** Characteristics of clinical trials on FMT for CD.

References	Author (year)	Study type	NCT number	Country	Location	Behavior	Interventions	Adverse events	Primary outcomes	Evaluation indexes definition	Stool resource
[49]	Suskind (2015)	Cohort	NCT01757964*	America	22% L1, 11% L2, 67% L3, and 22% L4	100% B1	Nasogastric tube	Abdominal pain (n = 5), bloating (n = 5), diarrhea (n = 4), and stuffy nose (n = 1)	At week 2 clinical remission: 77.8% (7/9).	Clinical remission: PCDAI score < 10	Relatives
[50]	Wei (2015)	Cohort	NA	Asia	NA	NA	Colonoscopy or nasojejunal tube infusion	NA	NA	CDAI score decreased from 345.00 ± 77.78 to 135.00 ± 7.07 (*P* = .082)	Relatives or unrelated
[51]	Cui (2015)	Cohort	NCT01793831	Asia	27% L1, 10% L2, and 63% L3	NA	Input into mid-gut by a tube	Fever (n = 2) and diarrhea (n = 7)	At month 1 clinical improvement: 86.7% (26/30); clinical remission: 76.7% (23/30)	Clinical improvement: decrease in HBI score > 3 Clinical remission: HBI score ≤ 4.	Relatives or unrelated
[52]	Vermeire (2016)	Cohort	NA*	Europe	83% L3	NA	Nasojejunal or rectal tube	NA	None of the patients clinically improved with FMT	Simplified endoscopic activity score CD < 3 in 8 weeks	Relatives or unrelated
[53]	Vaughn (2016)	Cohort	NCT01847170*	North America	37% L2 and 63% L3	84%B1, 11% B2, 1% B3, and 32% P	Performed by colonoscopy	No adverse events were observed among the patients	At week 4 clinical response: 58%(11/19)	Clinical response: decrease in HBI score> 3	Unrelated
[54]	Goyal (2018)	Cohort	NCT02108821	North America	29% L3 and 71% L4	100% B1	Performed by colonoscopy and gastroscope	NA	At month 1 clinical response: 71%(5/7) At month 6 clinical response: 43%(3/7) Two patients achieved clinical remission at 6 months post-FMT	Response: decrease in 12.5 points in PCDAI at 1 month. Remission: fecal biomarkers return to normal and a PCDAI of 0 points	Relatives or unrelated
[55]	Wang (2018)	Cohort	NCT01793831	Asia	15% L1, 25% L2, 59% L3, and 1% L4	45% B1, 36% B2, and 19% B3	Delivered into the mid-gut through a nasojejunal tube or gastroscopic	Herpes zoster (n = 1), hematochezia (n = 1), bloating (n = 1), vomiturition (n = 1), increase flatulence (n = 2), abdominal pain (n = 4), fever (n = 8), and increase frequency of defecation (n = 13)	At month 1 response: 71.2% (99/139) Clinical remission: 56.8% (79/139).	Clinical response: decrease in HBI score>3 Clinical remission: HBI ≤ 4	Relatives or unrelated
[56]	Karolewska (2018)	Cohort	NA	Europe	100% L2	100% B1	Via gastroscopy or a nasoduodenal tube	NA	At days 18–33, all (2/2) patients achieved clinical response and clinical remission	Clinical response: decrease in PCDAI I score>15 Clinical remission: PCDAI score< 10	Unrelated healthy donor
[57]	Gutin (2019)	Cohort	NCT02460705*	North America	North America	67% B1 and 33% B2	Via colonoscopy	Abdominal pain (n = 2) and diarrhea (n = 2)	At month 1 clinical response: 30.0% (3/10) Clinical remission: 10.0% (1/10).	Clinical response: improvement in HBI ≥ 3 Clinical remission: improvement in HBI < 3	Unrelated healthy donor
[58]	Yang (2020)	RCT	NA*	Asia	100% L2	NA	Via gastroscopy or colonoscopy	Nausea (n = 1), reflux (n = 4), belching (n = 2), diarrhea (n = 10), constipation (n = 1), fever (n = 2), abdominal pain (n = 5), and abdominal distention (n = 3)	At week 2 clinical response: 77.8% (21/27) Clinical remission: 66.7% (18/27)	Clinical response: improvement in CDAI > 100 Clinical remission: improvement in CDAI < 150	Relatives or unrelated
[59]	Xiang (2020)	Cohort	NCT01793831	Asia	17% L1, 17% L2, 58% L3, and 9% L4	44% B1, 39% B2, 17% B3, and 17% P	Via nasojejunal tube, mid-gut TET, colonic TET, and gastroscopy	No long-term (>1 month) FMT-related AEs were observed	At month 1 clinical response:75.3% (131/174)	Clinical remission: HBI ≤ 4 Clinical improvement: decrease in HBI score > 3 The clinical response included clinical improvement and clinical remission	Relatives or unrelated
[60]	Osaki (2021)	Cohort	NA*	Asia	25% L1 and 75% L3	25% B1, 50% B2, 25% B3, and 100% P	Via antegrade balloon-assisted enteroscopy	There were no serious adverse events in any patients within 24 weeks after FMT	At week 8 clinical response:100.0% (4/4)	Clinical response: decrease in CDAI score > 70	
[61]	Xiang (2021)	Cohort	NCT02897661	Asia	1% L1, 42% L2, 68% L3, and 11% L4	26% B1, 37% B2, 37% B3, and 47% P	Via gastroscope or mid-gut TET tube	Abdominal pain (n = 2), diarrhea (n = 1), and pericoronitis (n = 1)	At day 15 clinical remission 78.9% (11/19)	Clinical remission: Mayo score ≤ 2 points and complete mucosal healing (Mayo endoscopy sub score ≤ 1)	Unrelated healthy donor

B1 = inflammatory, B2 = structuring, B3 = penetrating, HBI = Harvey-Bradshaw index, L1 = small intestine, L2 = colonic disease, L3 = ileocolonic disease, L4 = upper gastrointestinal tract, NA = not available, NCT = National Clinical Trials, P = perianal disease modifier, PCDAI = pediatric Crohn disease activity index, RCT = randomized controlled trial, TET = transendoscopic enteral tubing.

* Data of gut microbiome to FMT therapy were available for discussion, respectively.

## 3. Changes in microbial characteristics in CD

Ecological dysbiosis plays both a causal and consequential role in CD, a chronic IBD. Research has revealed significant alterations in the gut microbiota of patients with CD, with dysbiosis being more pronounced compared with patients with UC.^[19,62]^ Factors such as disease site (ileocecal or colonic) and surgery (ileocecal defect) are also important confounding variables.^[34,62]^ The establishment of a normal commensal microbial population begins after birth through vaginal delivery and reaches relative stability during puberty.^[36]^ However, the use of antibiotics, sanitation practices, dietary differences, and vaccinations can significantly impact the microbiota composition.^[36,63]^ In a study by Ng et al,^[64]^ 1 month after the second dose of the Coronavirus disease 2019 vaccine, a large number of species in the intestinal microbiota showed a decrease in biodiversity. The effects of vaccination on the gut microbiota are often reciprocal.^[65]^ Finally, the choice of sequencing technologies (PCR, 16S rRNA sequencing, and shotgun metagenomics), annotation databases, and analytical methods can influence the assay results.^[66]^ This article provides a summary of gut microbial characteristics in patients with CD based on population-based studies obtained through searches on PubMed, Embase, and Cochrane databases (Table 1).

### 3.1. Alterations in gut microbial diversity

Microbial diversity encompasses the abundance and variety of microbial species within a specific ecosystem. The human gut represents a microecosystem where the diversity of its microbiota plays a crucial role in regulating normal physiological functions and intestinal immunity. Comparisons between patients with CD and healthy individuals in terms of gut microbial diversity typically involve α-diversity and β-diversity. The α-diversity assesses the species richness and evenness in a specific region, with commonly used metrics including the Chao1 richness estimator, the Shannon-Wiener diversity index, and the Simpson diversity index. The β-diversity, on the other hand, focuses on the variation in species composition between different regions and involves analytical methods such as distance calculation between samples (Jaccard, Bray-Curtis, and Unifrac), principal co-ordinates analysis, and principal component analysis. According to a systematic review,^[66]^ 12 studies on CD were summarized, revealing that 9 of them reported lower α-diversity in patients with CD compared with healthy controls. Furthermore, the α-diversity of the gut microbiota tends to decrease with CD disease activity, while β-diversity is also influenced by the severity of the disease.^[67,68]^ In terms of β-diversity, healthy individuals were observed to cluster more closely together compared with those with IBD.^[69]^

### 3.2. Changes in the composition and abundance of gut microbiota

The human gut microbiota is comprised of >10 billion microorganisms and possesses 100× more genes compared with the human genome.^[54,70]^ Extensive research has consistently demonstrated the close association between intestinal microbiota and human health.^[71–73]^ Specifically, it is crucial in maintaining immune homeostasis within the intestinal mucosa.^[74]^ Studies have consistently shown significant differences in the intestinal microbiota and metabolite composition between patients with CD and healthy individuals. Consequently, it can be deduced that the dysregulated microecological environment in the intestines of patients with CD is closely linked to metabolic disorders and the development of CD.^[75]^ Most studies have yielded a similar consensus at the phylum level, indicating an increase in Proteobacteria and a decrease in Firmicutes and Bacteroidetes.

#### 3.2.1. Decrease in “beneficial bacteria” in CDs

In the investigation of gut microbiota with protective effects against CD, *Faecalibacterium prausnitzii*^[20,27,29,39–41,62,76,77]^ and *Roseburia*, particularly *Roseburia intestinalis*,^[17,21,24,34,62]^ have emerged as organisms of significant interest within the thick-walled bacteria phylum. Multiple studies have consistently reported a notable decrease in their abundance among patients with CD, with the exception of one study that observed an elevated level of *F prausnitzii* in children with new-onset pediatric CD,^[42]^ a finding that was subsequently challenged by another study.^[43]^ The latter study concluded that *F prausnitzii* was increased in children with new-onset IBD characterized by CD but not in those with UC. Likewise, several Bacteroides species, including *Bacteroides uniformis*^[76]^ and *Bacteroides thetaiotaomicron*,^[23]^ exhibited similar declines in CD. The pathogenesis of CD is often associated with reduced levels of intestinal bacterial metabolites, such as short-chain fatty acids (including acetate, propionate, and butyrate), secondary bile acids, kynurenic acid, and an increase in indole and indole derivatives in the tryptophan metabolite.^[78]^

Within the *Bacteroides* genus, *B uniformis* has been identified as a significant producer of butyric acid, one of the short-chain fatty acids.^[76]^ Furthermore, in the *Clostridium* genus, there is evidence demonstrating that *Clostridium leptum*^[18,38,41]^ and *Clostridium lavalense*^[76]^ are notably reduced in patients with CD. *C leptum* is known to participate in carbohydrate fermentation and secondary bile acid production.^[79]^ Similarly, *C lavalense* is recognized as an important producer of butyrate.^[76]^ In addition, certain bacteria belonging to the Firmicutes phylum, such as *Eubacterium*,^[17,76]^ *Eubacterium rectale*,^[20,28]^ *Coprococcus*,^[17,21,24,32,33]^ *Anaerostipes*,^[19,21,35]^ and *Ruminococcus*^[17,24,34,76]^ from Firmicutes, *Actinobacillus*^[24]^ from Proteobacteria, and *Akkermansia* from Verrucomicrobia^[17]^ and especially *Akkermansia muciniphila*,^[32,35,47]^ have exhibited significant decreases in CD compared with healthy controls. Many of these bacteria are believed to have a protective role in the development of intestinal mucosal inflammation.

#### 3.2.2. Increase in “harmful bacteria” in CDs

*Bacteroidota fragilis* from Bacteroidota,^[62]^ *Ruminococcus gnavus*^[24,26,27,31,35]^ from Firmicutes, *Escherichia*^[17,19,24,30,32,62]^ from Proteobacteria, especially *Escherichia coli*,^[24,40,41,62]^ and *Fusobacterium*^[19,24,45]^ from Fusobacteria, especially *Fusobacteria nucleatum*,^[48]^ are bacteria that are often thought to be increased in patients with CD. *B fragilis* is an extensively studied anaerobic bacterium and one of the most frequently isolated species from clinical samples. It can be classified into nontoxigenic *B fragilis* (NTBF) and enterotoxigenic *B fragilis* (ETBF) based on the production of *B fragilis* toxin (BFT), which has been identified as a cause of diarrhea in patients and is possibly linked to the development of IBD.^[80]^ Numerous studies have consistently shown a significant reduction of *R gnavus*^[24,26,27,31,35]^ in patients with CD compared with healthy controls, making *R gnavus* a potential causative agent for CD.^[81]^ *Escherichia*, particularly *E coli*, is considered one of the most adaptable and pathogenic bacterial organisms.^[82]^ Pathogenic adherent invasive *E coli* (AIEC) has emerged as a leading candidate bacterial trigger in CD research and has been identified as an important risk factor for CD.^[83]^ *F nucleatum* is an anaerobic symbiotic bacterium that plays a crucial role in bridging early and late colonizers in the gut.^[84]^ It has been implicated as a pathogenic agent associated with the etiology of IBD, particularly CD.^[48]^

#### 3.2.3. Changes in the abundance of some microbiota are still controversial in different studies

The abundance of *Bacteroides*^[18,22,24,41,76]^ from Bacteroidota, *Lactobacillus*^[35,37,41,45]^ from Firmicutes, and *Bifidobacterium*^[34,41,62,76]^ from Actinobacteria remains somewhat controversial across different studies. For instance, a study conducted in Romania^[41]^ comparing the intestinal microbiota of adults with CD and healthy controls found a higher abundance of *Bacteroides* in patients with CD, while a study conducted in Iran^[22]^ reported a significant decrease in *Bacteroides* abundance in patients with IBD, particularly in underweight patients with CD compared with those with UC. Similarly, in a study comparing the fecal and intestinal mucosal microbiota of patients with CD and healthy individuals, an enrichment of *Lactobacillus spp* was observed in patients with CD’ fecal samples and intestinal mucosa.^[45]^ Similar findings were reported in another study,^[41]^ with the differences being particularly significant in patients with CD with ileocolonic disease. However, 2 other studies reached the opposite conclusion.^[35,37]^ The relative abundance of *Bifidobacterium* was found to be either elevated^[41,76]^ or decreased^[34,62]^ in patients with CD compared with healthy controls. The relative abundance of *Bifidobacterium* seems to be associated with the disease state of CD,^[85]^ with higher abundance observed in active CD compared with healthy controls or patients with CD in remission, which could explain the observed discrepancies.

## 4. Possible mechanisms for the role of specific gut microbes in the development of CD

To enhance our understanding of the involvement of intestinal microorganisms in CD, this study offers an overview of the microbiota that potentially contribute to a “protective” or “pathogenic” role in CD, along with their respective mechanisms of action. These mechanisms have predominantly been investigated and elucidated using animal models of colitis induced by dextran sodium sulfate (DSS) or 2,4,6-trinitrobenzenesulfonic acid (TNBS).

### 4.1. Microorganisms that may play a “protective” role

#### 4.1.1. F prausnitzii

*F prausnitzii* represents >5% of the human gut bacteria and is renowned for its prominent role in maintaining human health.^[86]^ It possesses anti-inflammatory properties, as demonstrated in a TNBS-induced mouse model of colitis, where it hinders nuclear factor-kappa B (NF-κB) activation and interleukin (IL)-8 production through the release of metabolites. Meta-analysis and systematic evaluations have consistently indicated a negative association between *F prausnitzii* and CD, suggesting its potential protective role in CD development.^[87,88]^ In fact, studies have confirmed that the production of butyrate by *F prausnitzii* contributes to the preservation of Th17/regulatory T (Treg) balance and amelioration of intestinal inflammation by inhibiting histone deacetylase 1.^[89]^ Among the 3 effector CD4^+^ T-lymphocyte subsets (Th1, Th2, and Th17 cells) that can contribute to the initiation and progression of IBD,^[90]^ Treg cells play a crucial role in inhibition. Treg cells suppress the Th17 response and counteract inflammation in the colon by downregulating the Th1 response via transforming growth factor-beta (TGF-β).^[91]^ Unfortunately, patients with IBD often exhibit reduced Treg cell levels, contributing to increased inflammation.^[92]^ These Treg cells normally produce IL-10 and serve to regulate the secretion of pro-inflammatory IL-17 and interferon-γ by effector T cells.^[93]^ *F prausnitzii*, through the production of butyrate, exerts anti-inflammatory effects by inhibiting the IL-6/signal transducer and activator of transcription 3 and IL-17 pathways while also promoting the expression of forkhead box protein P3 in rodent colorectal colitis.^[89]^ Moreover, *F prausnitzii* produces a 15-kDa protein known as a microbial anti-inflammatory molecule, which suppresses the NF-κB pathway in intestinal epithelial cells (IECs), further contributing to its anti-inflammatory effects.^[94]^ Recent studies have indicated that this microbial anti-inflammatory molecule is capable of restoring the structure and function of the intestinal barrier in diabetic conditions through the modulation of the tight junction pathway and zona occludens-1 expression.^[95]^

#### 4.1.2. R intestinalis

*R intestinalis* is likewise one of the main producers of butyrate,^[96]^ which is found in reduced amounts in patients with CD. In a mouse model of colitis induced by DSS and TNBS, the supernatant of *R intestinalis* demonstrated an anti-inflammatory effect by reducing the population of inflammatory macrophages and Th17 cells in the colon while downregulating the expression of IL-6 and signal transducer and activator of transcription 3.^[97]^ It has been observed that flagellin, a key structural component of *R intestinalis*, may exert anti-inflammatory effects by inducing the upregulation of long noncoding RNA (hypoxia-inducible factor 1A antisense RNA 2).^[98]^ Toll-like receptor 5, downregulated in the IECs of mice with DSS-induced colitis, can be upregulated by *R intestinalis* through its production of butyrate and activation by *R intestinalis* flagellin. This induction prompts IECs to secrete IL-10 and TGF-β, thereby exerting anti-inflammatory effects.^[99]^ The inhibitory effect of *R intestinalis* on the development of CD is attributed to its promotion of Treg differentiation. Specifically, *R intestinalis* stimulates the production of thymic stromal lymphopoietin in intestinal IECs via toll-like receptor 5, which facilitates the differentiation of anti-inflammatory Tregs by triggering the secretion of IL-10 and TGF-β from monocyte-derived dendritic cells.^[100]^ In a rat model of colitis, Xiao et al^[101]^ demonstrated that the use of *R intestinalis* as a probiotic, targeted to the intestinal tract through wireless magnetic iron oxide nanoparticles, resulted in favorable therapeutic efficacy and improved diversity of the intestinal microbiota.

#### 4.1.3. A muciniphila

*A muciniphila*, a commensal bacterium that resides in the intestinal mucosal layer, possesses notable metabolic and anti-inflammatory properties, making it a promising candidate for probiotic use.^[102,103]^ One beneficial trait of *A muciniphila* is its ability to adhere to the mucus layer.^[104–107]^ The mucosal layer in the intestine serves to protect epithelial cells from microbial attacks while also providing a source of energy for microorganisms that utilize them as nutrients. Insufficient levels of *A muciniphila* in the intestinal tract can result in thinning of the mucosa, leading to a weakened intestinal barrier that facilitates the invasion of toxins into the host.^[102]^ The specific mechanism of action may involve the binding of the outer-membrane protein Amuc_1100 in *A muciniphila* to toll-like receptor 2 on the surface of IECs. This leads to the activation of the adenosine monophosphate-activated protein kinase pathway, increased expression of the zonula occludens-1 protein (which functions as a backbone linker), and inhibition of Claudin2, a gene responsible for generating intestinal permeability.^[106,108]^ A study by Kang et al^[109]^ demonstrated that extracellular vesicles produced by *A muciniphila* also regulate intestinal immunity and homeostasis in vivo, providing protection against dextrose sodium sulfate-induced colitis in mice. Furthermore, *A muciniphila* exhibits anti-inflammatory effects. *A muciniphila Muc^T^* was initially observed to enhance the expression of the antimicrobial peptide regeneration-derived protein III gamma in the intestinal tract, thereby restoring the thickness of the mucus layer and promoting direct bactericidal activity against gram-positive bacteria.^[105]^ In addition, *A muciniphila* bacteria suppress the activation of the inflammatory signaling pathway NF-κB by activating toll-like receptor 2 on the surface of IECs. This, in turn, reduces the expression of inflammatory factors, such as tumor necrosis factor α and IL-1, while increasing the expression of anti-inflammatory factors, including TGF-β and IL-10.^[108]^

#### 4.1.4. Nontoxigenic *B fragilis*

NTBF, in contrast to ETBF, establishes a symbiotic relationship with the host organism. Studies have indicated that NTBF possesses anti-inflammatory properties in the colons of mice with Helicobacter hepaticus–induced colitis.^[110]^ In addition, NTBF competitively inhibits the attachment of ETBF through type VI secretion, effectively decreasing toxin release and safeguarding the host from intestinal inflammatory diseases.^[111]^ It has been reported that the NTBF strain may also exhibit probiotic characteristics. Apart from maintaining intestinal homeostasis, the delivery of beneficial molecules through Polysaccharide A and other outer-membrane vesicles may positively impact intestinal health.^[112]^ Interestingly, a study discovered that the abundance of NTBF increases as the immune system gradually develops in children between the ages of 1 and 2 years.^[113]^ Furthermore, NTBF suppresses the nucleotide-binding oligomerization domain, leucine-rich repeat and pyrin domain-containing protein 3-mediated inflammatory signaling pathway by producing butyrate, which inhibits macrophage activation and reduces the release of pro-inflammatory mediators such as IL-18 and IL-1β. This reduction in the level of intestinal mucosal inflammation aims to mitigate the inflammatory response.^[114]^

### 4.2. Microorganisms that may play a “pathogenic” role

#### 4.2.1. Enterotoxigenic *B fragilis*

ETBF is the most frequently isolated anaerobic bacterium in cases involving diarrhea, peritonitis, intra-abdominal abscesses, and sepsis and is strongly linked to active.^[115,116]^ Its secretion of the virulence factor called BFT is a crucial contributor to intestinal inflammation in both human and animal models. The toxin induces cleavage of E-calmodulin, secretion of IL-8, and epithelial cell proliferation.^[117,118]^ The cleavage of E-calmodulin may be associated with an unidentified receptor binding on both BFT and IECs.^[116]^ The stimulation of HT-29 cells by BFT leads to IL-8 secretion via activation of the G protein–coupled receptor 35.^[119]^ BFT also prompts the nuclear localization of β-linker proteins by cleaving E-calmodulin, causing the transcription and translation of c-myc proto-oncogene and consequent sustained proliferation of intestinal mucosal epithelial cells.^[120]^ Preclinical studies have demonstrated that BFT induces persistent colitis in specific pathogen-free C57BL/6 wild-type mice and even rapid lethal colitis in germ-free mice.^[117]^

#### 4.2.2. Adherent invasive *E coli*

While the majority of *E coli* strains are regarded as harmless, there exist 6 known pathogenic strains: enteropathogenic *E coli*, Shiga toxin-producing *E coli*, enterotoxigenic *E coli*, enteroaggregative *E coli*, enteroinvasive *E coli*, and diffusely adherent *E coli*.^[121]^ Studies correlating these strains with IBD indicate that AIEC is associated with CD, while diffusely adherent *E coli* is linked to UC. In investigations on CD pathogenesis, AIEC exerts its effects on protein synthesis, signal transduction, ion secretion, cytokinesis, gene transcription, cytoskeletal function, and mitochondrial function within host cells. AIEC is able to survive in macrophages, induces the production of TNF-α, and promotes granulomatous inflammatory responses.^[122]^ In a prospective cohort study involving patients with CD, colonization by AIEC was observed to be associated with an increased presence of *R gnavus*, another bacterium deeply implicated in the development of CD.^[123]^ Nonetheless, the in vivo pathogenesis of AIEC and any specific genes pertaining to AIEC remain poorly understood, necessitating further research to elucidate the precise mechanisms underlying its pathogenicity.

#### 4.2.3. R gnavus

*R gnavus*, a strictly anaerobic bacterium, was initially isolated from the human gastrointestinal tract in 1974.^[124,125]^ It has been implicated in causing symptoms of CD due to its production of a glucorhamnan polysaccharide, which efficiently prompts dendritic cells to secrete the inflammatory cytokine TNF-α. This secretion is reliant on toll-like receptor 4.^[53,81]^ A gut microbiota analysis encompassing 68 patients with CD, 84 unaffected relatives, and 55 matched healthy controls confirmed and quantified differences in microbiota using real-time fluorescence quantitative PCR. The findings revealed that *R gnavus* was the sole bacterial species associated with dysbiosis that exhibited an increased abundance in CD.^[39]^ Another study involving hundreds of patients with cancer undergoing hematopoietic cell transplantation discovered a negative correlation between the absolute abundance of *R gnavus* and lymphocyte ratios during the recovery phase following chemotherapy and stem cell implantation. This suggests that *R gnavus* may contribute to high neutrophil-to-lymphocyte ratios, which are commonly associated with poor outcomes in IBD.^[55]^

## 5. Clinical application of FMT in CD

Clinical trials investigating the use of FMT for the treatment of IBD have predominantly focused on UC, with a notable lack of research dedicated to CD.^[78]^

Therefore, our review specifically examines FMT studies^[52,57,58,69,126–134]^ (13 in total) that have explored the efficacy (clinical response rate and clinical remission rate), safety (AEs), and impact on the intestinal microbiome of FMT in treating active CD.

### 5.1. Outcomes of FMT for CD

#### 5.1.1. Definition of clinical end points for CD

The clinical response in CD is commonly evaluated using the CD activity index^[135]^ and the Harvey-Bradshaw index.^[127,128,130,131]^ Other metrics, such as the simple endoscopic score for CD, C-reactive protein, and blood sedimentation, have also been employed in certain clinical trials to assist in measuring efficacy.^[52,130,136]^

#### 5.1.2. Factors affecting clinical end points

Several factors have been identified as influencing the efficacy of FMT. These include donor selection, graft route, microbial state (fresh/frozen), and recipient status, which are all recognized as crucial elements for successful graft outcomes. The Nanjing Consensus,^[137]^ led by Zhang et al in China, provides an extensive description of the rigorous donor selection process. In addition, the consensus introduces the concept of washed microbiota transplantation (WMT) as an enhancement to traditional FMT. WMT involves repeated washing of the bacterial microbiota to eliminate harmful substances, thus improving therapeutic efficacy and minimizing adverse effects.^[138]^

The initial stage of FMT involves the selection of a suitable donor. Previous research has indicated that the composition and diversity of the donor species may serve as a predictive factor for the therapeutic effectiveness of FMT in treating IBD. In addition, the identification of FMT superdonors has been reported.^[40]^ A Belgian study further supports the notion that a higher abundance of donor species is associated with successful outcomes in FMT-based IBD treatments.^[52]^ Notably, clinical responders in 2 separate studies demonstrated a more substantial shift toward the donor’s microbiome after FMT compared with nonresponders.^[69,127]^ To ensure an ample supply and minimize the risk of administering ineffective donor feces, the use of multidonor FMT infusions is recommended.^[78]^ It is important to note that the safety and efficacy of FMT are not only influenced by the microbiome but also by the pathway through which the intervention is performed.^[60]^ The pathways for the reconstruction of the intestinal microecology can be categorized into 3 areas: the upper, middle, and lower gastrointestinal tracts.^[49,137,139]^ The delivery of FMT can be carried out through various routes, namely, the upper, middle, and lower gastrointestinal routes.^[25]^ The upper gastrointestinal route includes traditional methods such as nasogastric tube infusion and oral-fecal capsules. The middle gastrointestinal route involves the use of a nasojejunal tube, endoscopic middle gastrointestinal tube implantation, or gastroscopic forceps orifice route. The lower gastrointestinal routes encompass enemas, enteroscopy, colostomy, and transendoscopic enteral tubing. Among these, colonic transendoscopic enteral tubing has emerged as a novel, convenient, painless, and reproducible approach for delivering WMT, ensuring high patient satisfaction.^[4]^ A meta-analysis conducted on FMT for CDI revealed that the lower gastrointestinal route appeared to be more effective in preventing CDI recurrence compared with the upper gastrointestinal route.^[44]^ Similarly, in a meta-analysis of FMT for CD involving 15 studies,^[46]^ FMT delivered via the upper gastrointestinal route initially exhibited higher rates of early efficacy (ranging from 75% to 100%). However, this difference could not be maintained during the follow-up period beyond 8 weeks.

Furthermore, it was determined in the study that the dosage of FMT and the state of the bacterial fluid (fresh or frozen) did not affect clinical outcomes. However, another meta-analysis indicated that the clinical remission rate of fresh fecal FMT was higher compared with frozen fecal FMT (73% vs 43%; *P* < .05).^[50]^ While donor factors have received substantial attention, the clinical status of recipients prior to FMT has received less focus, despite potentially being a significant factor in treatment response and efficacy.^[51]^ In the therapy of CDI, serious and complex indications have been observed to strongly influence FMT outcomes.^[56]^ However, there are insufficient research data establishing a relationship between the efficacy of FMT and the severity of CD. Thus, a systematic trial investigating the efficacy of FMT in relation to CD severity is warranted. Previous studies have demonstrated a positive correlation between specific gut microbial characteristics of recipients and the outcomes of FMT treatment (Table 4). Responders were found to have a higher abundance of *Enterobacteriaceae* and *Bifidobacterium* members, while nonresponders displayed an elevated relative abundance of members from *Lachnospiraceae* and *Ruminococcaceae*.^[130]^

### 5.2. AEs of FMT in CD treatment

A systematic review encompassing 129 studies on FMT for the treatment of various diseases reported an overall incidence of AEs at 19%.^[59]^ Within the scope of 13 FMT clinical trials analyzed in this review, the overall incidence of AEs was found to be 35.0% (90/257), with detailed information on AEs available for a total of 257 patients with CD. Gastrointestinal symptoms such as abdominal pain, diarrhea, and bloating were identified as more prevalent, accounting for 18.4%, 40.0%, and 12.6%, respectively, as presented in Table 3. Among the extraintestinal symptoms, fever was the most frequently reported, comprising 13.8% of all AEs, followed by pericoronary arthritis, nasal congestion, and herpes zoster. It is important to note that 95.6% (86/90) of the recorded AEs were categorized as mild, resolving spontaneously within a short duration of time.

**Table 3:** Adverse event data of FMT in CD.

Type	References	Patients	MAEs, n (%)	SAEs, n (%)
Diarrhea	[49,51,55,57,58,61]	37	35 (40.7)	2 (50.0)
Abdominal pain	[49,55,57,58,61]	18	16 (18.6)	2 (50.0)
Fever	[51,55,58]	12	12 (14.0)	0 (0)
Bloating	[49,55,58]	11	11 (12.8)	0 (0)
Reflux	[58]	4	4 (4.7)	0 (0)
Belching	[58]	2	2 (2.3)	0 (0)
Vomiturition	[55]	1	1 (1.2)	0 (0)
Nausea	[58]	1	1 (1.2)	0 (0)
Constipation	[58]	1	1 (1.2)	0 (0)
Pericoronitis	[61]	1	1 (1.2)	0 (0)
Stuffy nose	[49]	1	1 (1.2)	0 (0)
Hematochezia	[55]	1	1 (1.2)	0 (0)

MAE = minor adverse event, SAE = serious adverse event.

**Table 4:** Changes in gut bacteria in CD-related clinical trials.

References	Author (year)	α-diversity	β-diversity	Baseline donor vs patients*	Post-FMT vs pre-FMT*	Responders vs nonresponders*	Sequencing methods
[49]	Suskind (2015)	NA	NA	NA	NA	During clinical disease flare ↑*E coli*	Shotgun metagenomics
[52]	Vermeire (2016)	Increased bacterial OTUs in healthy donors compared with patients before FMT Higher number of OTUs in donors from whom the stools resulted in a successful FMT (*P* < .05)	NA	NA	Post-FMT (1 patient with CD improvement of symptoms) ↑Dialister, *Prevotella*, unclassified *Prevotellaceae*, and unclassified *Clostridiales*	NA	16 S rRNA sequencing
[53]	Vaughn (2016)	Responders increased Shannon index 4 and 8 weeks after FMT (*P* < .05)	No significant change between active CD and healthy donors The similarity of fecal samples between responders and donors (1 and 2 Bray-Curtis dissimilarity) was greater than that of nonresponders (*P* < .05) after FMT	Family level: pre-FMT ↑ in CD: *Veillonellaceae*, *Enterobacteriaceae*, *Pasteurellaceae*, and *Fusobacteriaceae*	NA	Post-FMT species-level ↑ in responders: *Bacteroides*, *Cellulosilyticus*, *Bilophila* unclassified, *D piger*, and so on ↑ in nonresponders: *Coprobacillus*, unclassified, *B massiliensis*, *R lactaris*, and so on	Shotgun metagenomics
[54]	Goyal (2018)	NA†	NA†	NA†	NA†	NA†	16 S rRNA sequencing
[57]	Gutin (2019)	Responders have lower baseline α-diversity before FMT The diversity of responders increasing more following FMT than nonresponders	Responders converging following FMT	NA	OTU level (genus) ↓*Bacteroides*, *Lachnospiraceae*_uncl, *Blautia*, *Collinsella*, *Dorea*, and *Coprococcus* in responders	OTU level pre-FMT ↑ in responders: *Enterobacteriaceae* and *Bifidobacterium* ↑ in nonresponders: *Lachnospiraceae* and *Ruminococcaceae* Post-FMT: ↑ in responders: *Lachnospiraceae* ↑ in nonresponders: *Ruminococcaceae* and *Bacteroides*	16 S rRNA sequencing
[58]	Yang (2020)	Shannon index increasing from 2.05 to 3.18 (*P* < .05); OTU increasing from 117 to 210 (*P* < .05)	NA	Patients with CD at the species level ↑*Clostridium*, *Cronobacter*, *Fusobacterium*, and *Streptococcus* genera ↓*Bacteroides*, *Eubacterium*, *Faecalibacterium*, *Roseburia*, and *Phascolarctobacterium* genera	NA	NA	16 S rRNA sequencing
[60]	Osaki (2021)	Increased Shannon index 8 weeks after FMT (NS)	No significant change	↓*Blautia*, *Dorea*, and *Eubacterium* counts	↑*Collinsella*, *Dorea*, and *Eubacterium* counts	NA	16 S rRNA sequencing

↑ = higher, ↓ = lower, NA = not available, NS = not significant, OTU = operational taxonomic unit.

* Taxonomy.

† The CD and UC cannot be accurately distinguished.

### 5.3. Changes in intestinal microbiota after FMT treatment in patients with CD

FMT primarily exerts its therapeutic effect by restoring the intestinal microecology of patients. This article provides a summary of the microbiological characteristics of FMT based on 6 of 13 clinical trials (refer to Table 4), with the goal of enhancing our understanding of the correlation between microbiota changes and efficacy. Five of these 6 studies demonstrated an increase in microbial diversity among patients receiving FMT^[52,127,130,131,133]^ although it remained lower than that of the donors. The increase in α-diversity was more pronounced in responders compared with nonresponders across all studies.^[52,127,130,131,133]^ Disease activity may influence specific microbiota changes, as evidenced by a US-based clinical study of FMT for CD where 2 patients experienced significant worsening of symptoms during the study. Longitudinal analysis of fecal metagenomics during these disease flare-ups revealed a notable rise in the relative abundance of *E coli*.^[57]^ In patients with CD, there is a reduction in the abundance of *Prevotella spp*,^[17,22,24]^ which increases transiently in patients with CD experiencing remission after FMT.^[52]^ Similarly, several other studies on FMT for UC have reported an increase in *Prevotella spp*.^[61,78]^ A study conducted in the United States^[127]^ revealed that patients with CD prior to FMT exhibited a significant increase at the family level in *Veillonellaceae*, *Enterobacteriaceae*, *Pasteurellaceae*, and *Fusobacteriaceae* at the family level compared with healthy donors. After FMT, the responding group displayed increases in species such as *Bacteroides cellulosilyticus*, *Bilophila* unclassified, and *Desulfovibrio piger*. In contrast, the nonresponder group was characterized by an increase in species such as *Coprobacillus* unclassified, *Bacteroides massiliensis*, and *Ruminococcus lactaris*. Another study by Gutin et al,^[130]^ conducted at the genus level, demonstrated a decrease in *Bacteroides*, *Lachnospiraceae_uncl*, *Blautia*, *Collinsella*, *Dorea*, and *Coprococcus* in the responder group after FMT compared with pretransplantation levels. When comparing the responding and nonresponding groups, a significant increase in *Lachnospiraceae* was observed in the responders after FMT, while the nonresponding group was primarily characterized by an increase in *Ruminococcaceae* and *Bacteroides*.

In the study by Yang et al,^[131]^ at the species level, there was a significant increase in the genera *Clostridium*, *Cronobacter*, *Fusobacterium*, and *Streptococcus* and a decrease in the genera *Bacteroides*, *Eubacterium*, *Faecalibacterium*, *Roseburia*, and *Phascolarctobacterium* in patients with CD compared with donors. Similarly, in the study conducted by Osaki et al,^[133]^ patients with CD showed lower counts of *Blautia*, *Dorea*, and *Eubacterium* prior to transplantation and elevated counts of *Collinsella*, *Dorea*, and *Eubacterium*, among others, after transplantation. On the whole, FMT may have a therapeutic role in optimizing the microbiota structure, increasing the abundance of beneficial bacteria, and reducing the colonization of pathogenic bacteria.

## 6. Discussion

Dysbiosis of the intestinal microbiota has been implicated in the etiology of CD, and the progression of intestinal inflammation in CD, in turn, affects the composition of the microbiota. In this review, we provided an overview of the characteristics of the CD microbiota at various taxonomic levels, including phylum, family, genus, and species. We also examined the detailed mechanisms of action of both “beneficial bacteria” and “harmful bacteria.” The interactions between these differential microbiota, which can be either protective or pathogenic for CD, are intricate. Not all results are consistent in different studies of the characteristic microbiota of patients with CD, for example, *Bacteroides* from Bacteroidota, *Lactobacillus* from Firmicutes, and *Bifidobacterium* from Actinobacteria remain highly controversial in different studies. There is still a need to design larger sample sizes and more rigorous experimental procedures to further clarify the role of flora in the development of CD.

FMT has emerged as a promising therapeutic approach for CD. However, compared with UC, FMT for CD has been relatively less studied, and its mechanism of action remains controversial. This article provided a summary of 13 clinical trials investigating FMT as a treatment for CD. It should be noted that certain IBD studies were unable to report separate data for CD and UC, resulting in the exclusion of some information related to efficacy, AEs, and colony analyses. Among the included studies, ≈54% (7 of 13; Tables 2 and 4) reported microbial analysis, with only 2 studies utilizing metagenomics sequencing to track species-level changes.^[57,127]^ Furthermore, only 1 study compared responders and nonresponders in CD.^[127]^ In addition, metabolomic testing before and after FMT was analyzed in only 1 of the 13 studies.^[133]^ Although further validation with larger populations and comprehensive analysis of clinical outcomes are warranted, the results of FMT demonstrate a significant correlation with specific microbial characteristics of both donors and recipients. As of now, our understanding of the efficacy and safety of FMT for CD is in its infancy. Moving forward, it is necessary to conduct larger sample studies and enhance the design and standardization of FMT procedures, encompassing donor screening criteria, transplantation routes, strain status, and strain preparation methods, to mitigate any potential biases. Furthermore, future research should explore additional genomics techniques (such as macroproteomics and culture genomics) to gain a deeper understanding of the dynamic alterations in the microbiota that are integral to FMT’s therapeutic effects and the underlying mechanisms of action.

## 7. Conclusion

There are significant differences between the intestinal microbiota of CDs and that of healthy individuals, mainly in the form of a decrease in certain “beneficial” bacteria and an increase in “harmful” bacteria. FMT may be an effective and relatively safe treatment for inducing clinical remission in patients with active CD, possibly by increasing the diversity of the intestinal microbiota and promoting the transfer of certain “beneficial” bacteria.

## Author contributions

**Conceptualization:** Shiju Chen, Daya Zhang, Da Li, Fan Zeng, Chen Chen, Feihu Bai.

**Data curation:** Shiju Chen, Daya Zhang, Da Li, Fan Zeng, Chen Chen, Feihu Bai.

**Formal analysis:** Shiju Chen, Daya Zhang, Da Li, Feihu Bai.

**Investigation:** Shiju Chen, Daya Zhang, Da Li, Feihu Bai.

**Methodology:** Shiju Chen, Feihu Bai.

**Project administration:** Shiju Chen.

**Resources:** Shiju Chen.

**Software:** Shiju Chen.

**Supervision:** Shiju Chen.

**Validation:** Shiju Chen.

**Visualization:** Shiju Chen.

**Writing – original draft:** Shiju Chen, Daya Zhang, Feihu Bai.

**Writing – review & editing:** Shiju Chen, Daya Zhang, Feihu Bai.

**Funding acquisition:** Feihu Bai.
